# Structure elucidation of β-cyclodextrin–xylazine complex by a combination of quantitative ^1^H–^1^H ROESY and molecular dynamics studies

**DOI:** 10.3762/bjoc.9.226

**Published:** 2013-09-23

**Authors:** Syed Mashhood Ali, Kehkeshan Fatma, Snehal Dhokale

**Affiliations:** 1 Department of Chemistry, Aligarh Muslim University, Aligarh-202002, India; 2 National Chemical Laboratory, Pune-411008, India

**Keywords:** β-cyclodextrin, inclusion complex, ROESY, simulation studies, xylazine

## Abstract

The complexation of xylazine with β-cyclodextrin was studied in aqueous medium. ^1^H NMR titrations confirmed the formation of a 1:1 inclusion complex. A ROESY spectrum was recorded with long mixing time which contained TOCSY artifacts. It only confirmed the presence of xylazine aromatic ring in the β-cyclodextrin cavity. No information regarding the mode of penetration, from the wide or narrow side, could be obtained. We calculated the peak intensity ratio from the inter-proton distances for the most stable conformations obtained by molecular dynamics studies in vacuum. The results show that highly accurate structural information can be deduced efficiently by the combined use of quantitative ROESY and molecular dynamics analysis. On the other hand, a ROESY spectrum with no spin diffusion can only compliment an averaged ensemble conformation obtained by molecular dynamics which is generally considered ambiguous.

## Introduction

The emergence and establishment of supramolecular chemistry as an important domain of science has fueled the development of complex chemical systems from components, interacting by non-covalent intermolecular forces. This field transcends the traditional barriers separating many disciplines of science and is the basis for most of the vital biological processes [1]. The basis of supramolecular chemistry is molecular recognition where host and guest species interact with each other and exist as a single system. These host–guest systems symbolize simplest examples of supramolecular systems in which a guest is encapsulated into the internal cavity of a larger host molecule. The most widely used hosts are cyclodextrins (CDs) which are crystalline, homogeneous and non-hygroscopic substances composed of α-1→4 linked glucose units. The outside surface of CDs is hydrophilic while the interior of the cavity is hydrophobic [2–3]. The encapsulation of a guest into the CD cavity has a profound effect on the chemical, physical and biological properties of the guest.

The structure establishment of CD inclusion complexes in solution state is a challenging task considering the fact that the inclusion phenomenon is a dynamic process, the included guest being in fast exchange between the free and bound state, and hence deriving instantaneous information with regard to structural changes is not easy.

NMR spectroscopy has evolved as a method of choice for studying inclusion complexes in solution, although only time-averaged structural information can be extracted in NMR time scale. Nuclear Overhauser Enhancement (NOE) experiments, which depend on internuclear distances, are used to identify the part of guest that is involved in complexation and to determine the position of the guest inside the CD cavity. However, the inability of NMR spectroscopy lies in the fact that it is not able to describe the mode of guest penetration, i.e. through the wide or the narrow rim side. The use of computational approaches along with NMR studies has proved to hold promise for systems which have dynamic nature [4]. The combination of these two techniques helps in understanding the complexation process and in gaining a deeper insight into the geometry of the system with a high degree of accuracy [5] and several cyclodextrin complexes have been investigated using this approach [6–7]. It has been observed that by the use of molecular dynamic techniques with no prior knowledge about the existence or geometry of CD inclusion complexes [8], accurate and reliable predictions can be made regarding the possible formation and other aspects of inclusion process by following a general simulation protocol [9].

**Figure 1:**
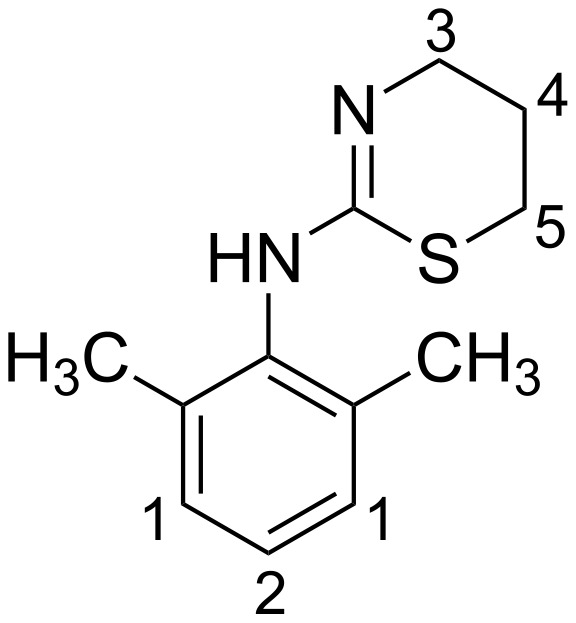
Structure of xylazine.

We report here the study of the complexation of β-CD with xylazine (XZ) (Figure 1) in aqueous medium by NMR spectroscopy and molecular dynamics. Xylazine has two rings, one substituted benzene ring which can only partially penetrate the CD cavity due to the presence of two methyl groups and a heterocyclic ring which should prefer aqueous medium outside the cavity. The study suggests that highly reliable structural information can be deduced by a combination of a poorly resolved ^1^H–^1^H ROESY spectrum, which otherwise gives only the information about the part of the guest included, and careful interpretation of molecular dynamics results in vacuum though it does not fully simulate the experimental conditions.

## Results and Discussion

### ^1^H NMR studies

Demarco and Thakkar first observed the chemical shift changes of the H-3’ and H-5’ protons, positioned inside the cavity of β-CD, in the presence of aromatic compounds which is inferred due to anisotropic effect of the aromatic ring entering the cavity [10]. Numerous studies ever since have shown that cavity protons and sometimes H-6’ also, located on the cavity rim at the narrow end, undergo appreciable shift changes upon inclusion of the guest while H-1’, H-2’ and H-4’, located outside the cavity, are relatively unaffected [11–14].

The ^1^H NMR spectral data of β-CD were in good agreement with the reported [15–16] and each proton resonance of xylazine, especially of the aromatic region, was assigned with the help of COSY data. The aromatic protons of xylazine were observed as a distorted doublet at 7.13 (2H, *J* = 9 Hz, H-1) and a doublet of doublet at 7.20 (1H, *J*_1_ = *J*_2_ = 9 Hz, H-2). Remaining protons were found resonating at 2.10 (H-4, -CH3) and 3.25 (H-3, 5).

**Figure 2:**
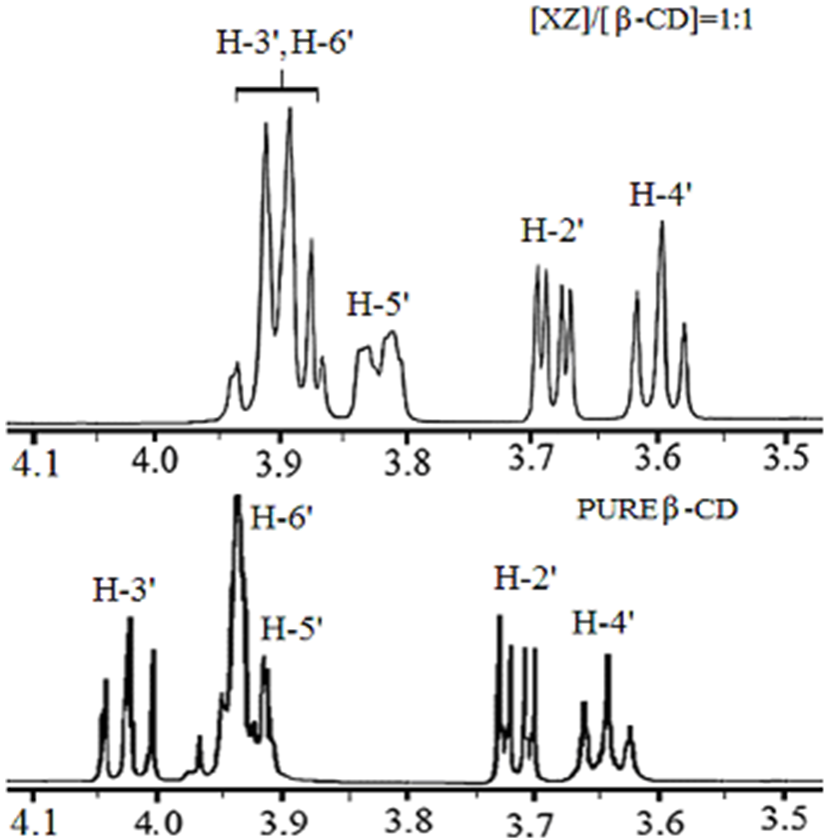
Comparative ^1^H NMR spectra of pure β-CD and a 1:1 β-CD–xylazine mixture showing β-CD regions.

^1^H NMR spectra of mixtures of xylazine and β-CD displayed upfield shift changes (Δδ), compared to pure β-CD, in the H-3’ and H-5’ proton resonances which were affected by concentration of xylazine. The magnitude of Δδ_H-3’_ was slightly larger than Δδ_H-5’_. Signals for H-2’ and H-4’ also slightly moved highfield but were unaffected by the change in concentration of xylazine (Figure 2). On the other hand, changes in shape and size of chemical shifts were observed for aromatic protons of xylazine but the magnitude of downfield shifts was relatively small. These observations confirmed the formation of the β-CD-xylazine complex.

### Stoichiometry

Of the various methods used to determine the stoichiometry and binding constant of host–guest complexes, ^1^H NMR titration experiments are most common. These titration experiments give Δδ data for many independent signals which can independently be used to determine the stoichiometry and binding constant. Several NMR versions of Benesi–Hildebrand equations [17] are used for this purpose and we have used Scott’s method [18]. The Scott’s equation gives the relationship between the apparent binding constant (*K*_a_), the complexation-induced shift at saturation (Δδ_s_), the guest or host concentrations (the species whose concentration is varied), and the observed shift change (Δδ_obs_). Scott’s equation for 1:1 β-CD–xylazine complex can be written as:

[1]
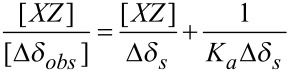


^1^H NMR spectra of pure β-CD, pure xylazine and their mixtures having molar ratios (β-CD: xylazine) 1:0.25, 1:0.50, 1:0.75, 1:1.00, 1:1.50 and 1:2.00 were recorded. NMR samples of mixtures were prepared by taking 9 mg of β-CD and adding a calculated amount of xylazine in 0.5 ml D_2_O.

**Figure 3:**
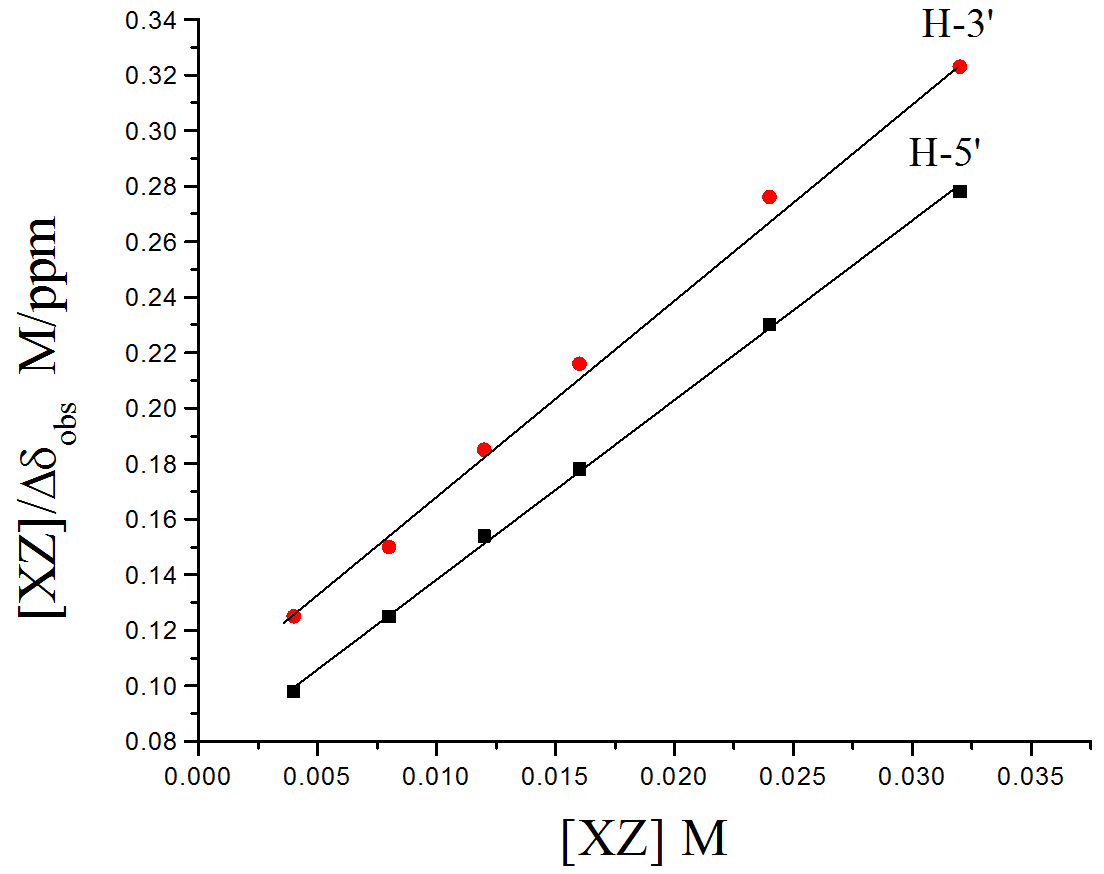
Scott’s plot of the chemical shift changes of β-CD cavity protons during titration with xylazine in D_2_O.

The Δδ_H-3’_ and Δδ_H-5’_ data was plotted in the form of [XZ]/Δδ_obs_ versus [XZ] (Figure 3) which gave linear fits confirming the 1:1 stoichiometry of the complex. The binding constant of the complex was calculated to be 86.45 M^−1^.

### ^1^H–^1^H ROESY studies

ROESY is an important tool for the study of large molecules and provides information on the through-space proximity of protons [19]. The host–guest interactions are displayed as intermolecular peaks between cavity protons and part of the guest involved in complexation. The strength of the crosspeak is proportional to the inverse sixth power of the distance between the interacting nuclei, *I* 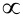 1/r^6^. However, for quantitative ROESY data analysis, one must understand the consequences of spin diffusion which occurs primarily for large molecules and long mixing times outside the “linear approximation” resulting in TOCSY artifacts. With a mixing time of around 0.2 s, TOCSY artifacts are generally not observed for large molecules (MW > 1200) but experiment requires several hours. On the other hand, ROESY spectrum of large molecules can be recorded in few minutes with long mixing time, e.g. 0.5s, but containing TOCSY artifacts.

**Figure 4:**
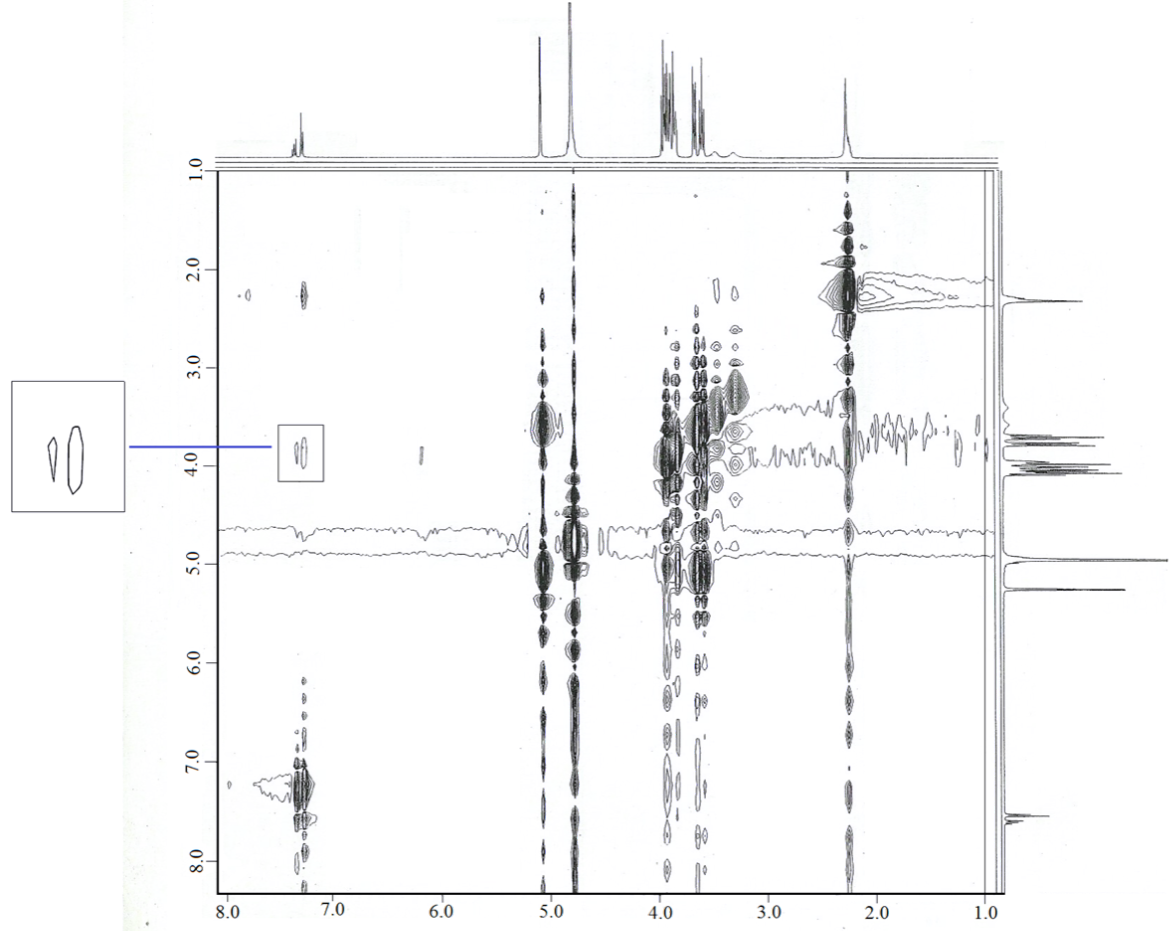
Full ROESY spectrum of a β-CD-xylazine 1:1 mixture showing intermolecular crosspeaks.

**Figure 5:**
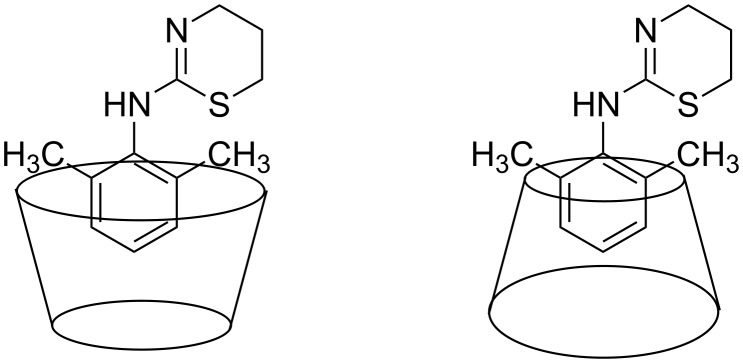
Two probable modes of the inclusion of xylazine into the β-CD cavity.

We recorded a ROESY spectrum of a 1:1 mixture of β-CD and xylazine with 0.5 s mixing time (Figure 4). The experiment was stopped as soon as the crosspeaks between xylazine and the cavity protons appeared. The region showing intermolecular peaks contained no TOCSY artifacts on one side of the diagonal but artifacts interfered with intermolecular peaks on the other side of the diagonal. The intermolecular crosspeaks between H-1 and H-2 of xylazine and the cavity protons confirmed the presence of an aromatic ring inside the β-CD cavity. It was found that the heterocyclic ring was not involved in the complexation. Also, no interactions were observed between the methyl groups of xylazine and the cavity protons suggesting partial penetration of the aromatic ring but whether the aromatic ring approached the cavity from the wide or narrow side was not clear. Thus, two geometries for the β-CD-xylazine complex can be assumed (Figure 5). We established the structure of the complex using this ROESY data and molecular dynamics simulation studies in vacuum. Earlier, we established the structure of a fexofenadine-α-CD complex [20] on the basis of molecular mechanics and ROESY data recorded with 0.5 s mixing time, but intermolecular peaks on both sides of the diagonal were clear in that case.

### Molecular dynamics

**Figure 6:**
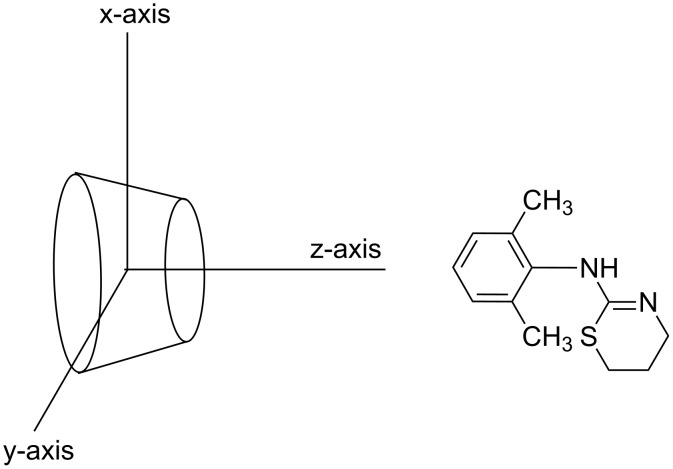
Coordinate system used to define the complexation process.

Molecular dynamics simulations for only aromatic ring were performed. All the calculations were performed using CS Chem3D Pro (Cambridge Soft Corp.) in vacuum at 298 K. Initial coordinates for β-CD were obtained from the Cambridge databank. The published X-ray coordinates for hydrated β-CD [21] were used as starting point after removal of the water molecule coordinates. The structure of xylazine was minimized to a RMS value of 0.1 kcal mol^−1^Å^−1^ using Allinger’s force field. Simulations were performed by placing the aromatic ring of xylazine near the mouth of the β-CD cavity along the z-axis either on the narrow (N) or wide (W) side (Figure 6).

**Figure 7:**
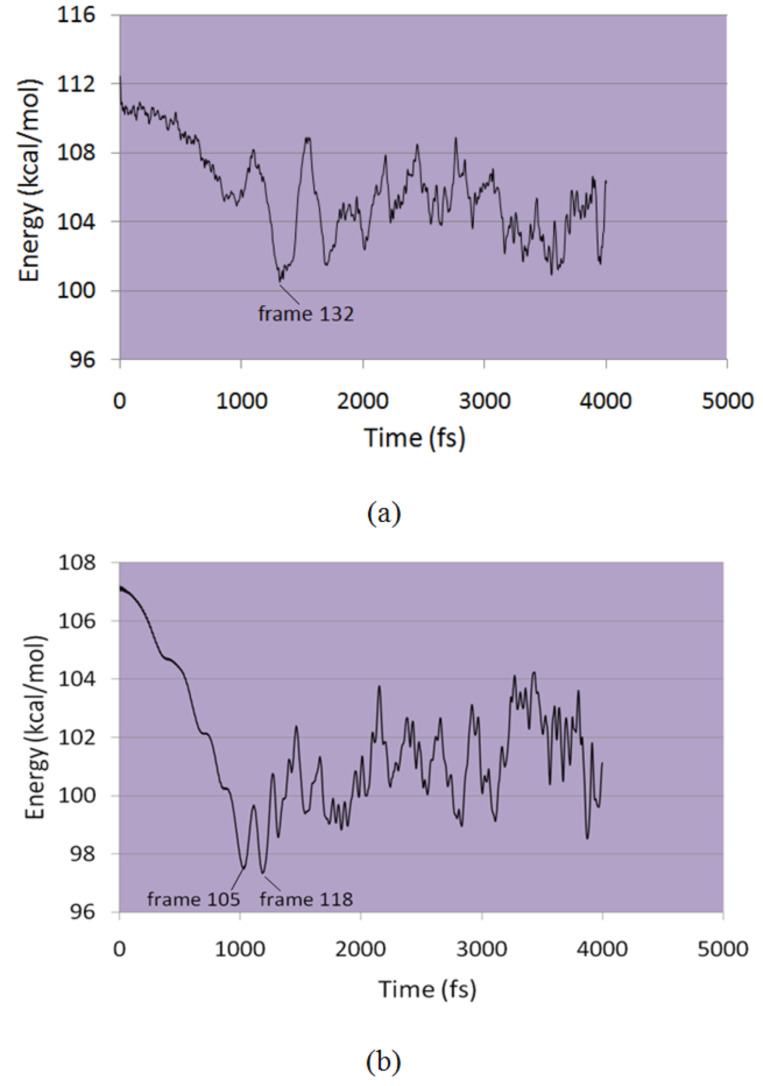
The time evolution of the potential energy calculated from the MD run in vacuum from (a) wide side and (b) narrow side of β-CD cavity.

The carbon skeleton of the β-CD was kept static but other atoms and the xylazine molecule were allowed to move. An iteration step of 1 fs was used and conformations were recorded after every 10 iterations with 4000 steps of equilibration. Molecular dynamics simulation results provided evidence that complexation of β-CD and xylazine is energetically favored. In both the cases, the total potential energy of the complex was less than the sum of the potential energies of the two components (Figure 7).

**Figure 8:**
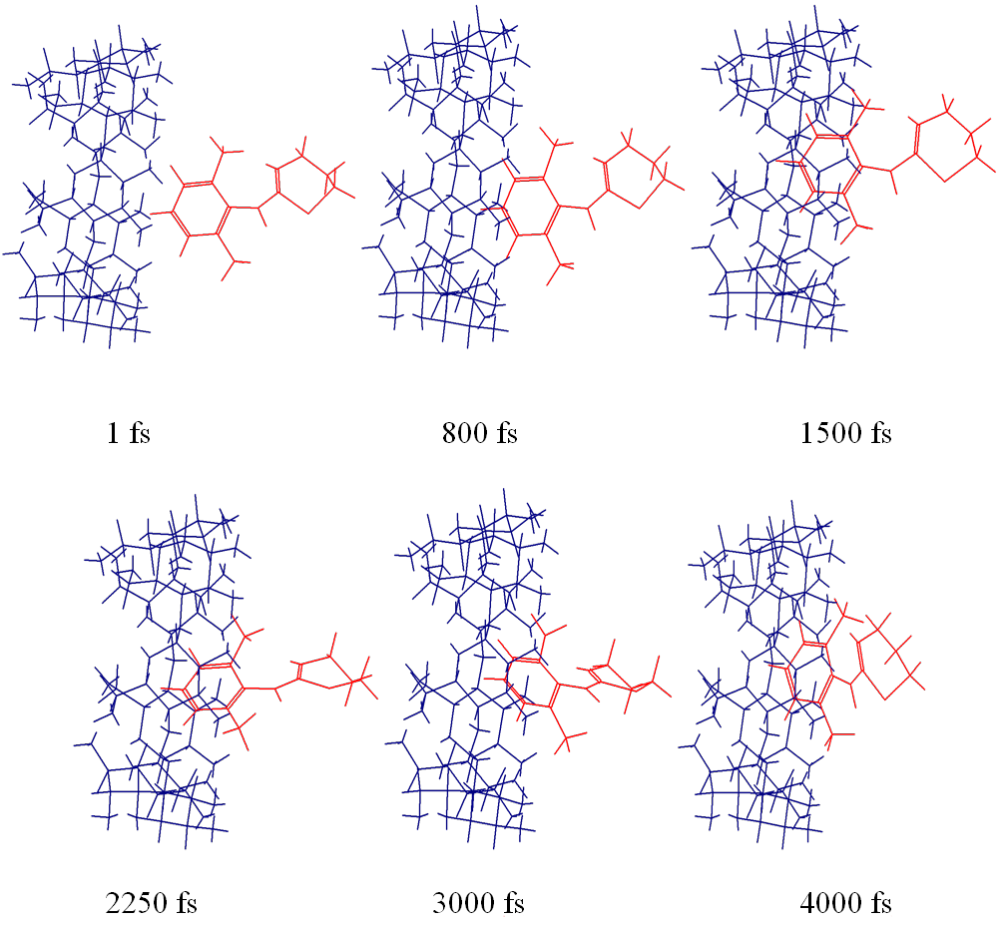
Snapshots showing the inclusion of xylazine into the β-CD cavity as obtained in the MD trajectory from wide side.

**Figure 9:**
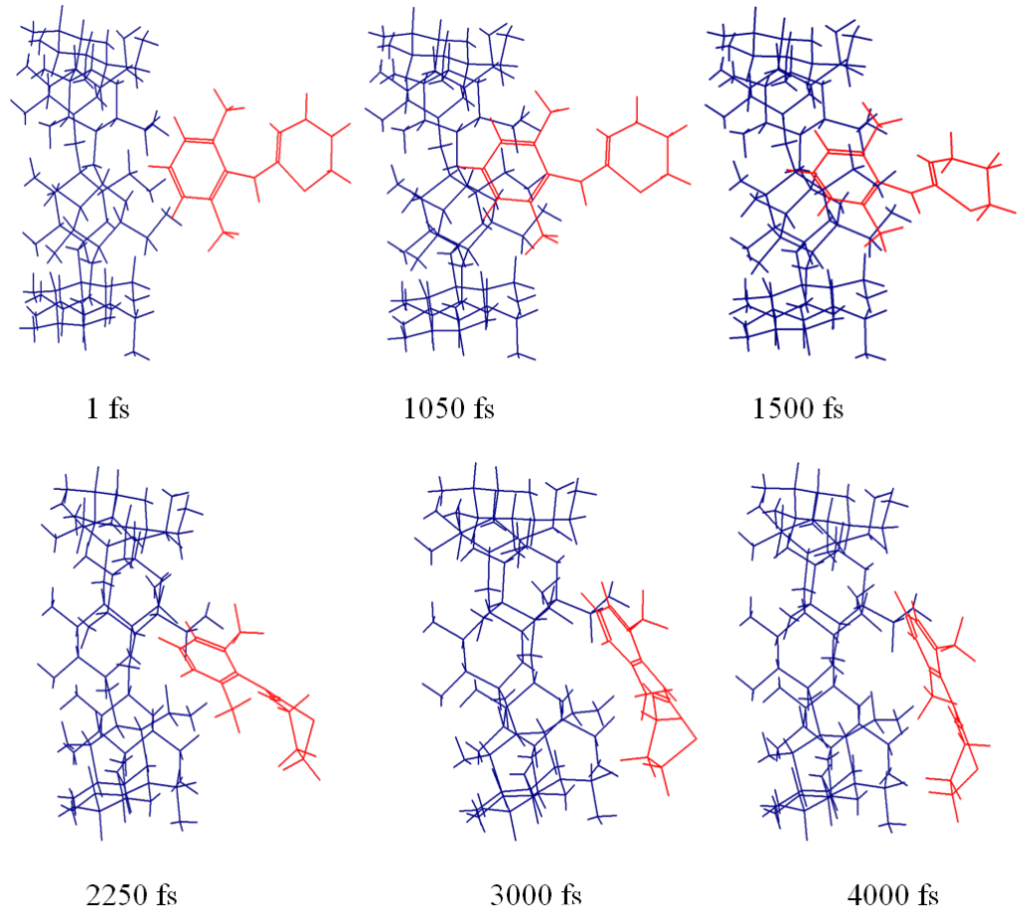
Snapshots showing the inclusion of xylazine into the β-CD cavity as obtained in the MD trajectory from narrow side.

The aromatic ring entered the cavity quickly and remained inside occupying various orientations by moving sideways, rotating or tilting, when xylazine was kept near the wider opening. The methyl groups always remained outside the cavity. In case of narrow side simulation, the aromatic ring also partially entered the cavity, with the methyl groups staying outside, but left the cavity after 2000 iterations and never returned. Figure 8 and Figure 9 show series of pivotal snapshots of the two simulation trajectories. ROESY peak intensity ratios were then calculated from the interproton distances of two frames from narrow side (frame 105 ,1050 fs, 97.83 kcal/mol; frame 118 , 1180 fs, 97.37 kcal/mol), and one frame from wide side (frame 132 , 1320 fs, 100.73 kcal/mol).

### Quantitative ^1^H–^1^H ROESY analysis

The ROESY peak intensities depend on the internuclear distances but are affected by several other factors and this is why the quantitative use of ROESY is generally avoided. Still there are numerous examples [22–24] where highly accurate structural information has been deduced by quantitative analysis of ROESY data. Macura and coworkers [25] and others [26] have shown that employing relative rather than absolute NOE intensities from within a given experiment can be used to calculate internuclear distances and vice versa with high accuracy using following equation,

[2]
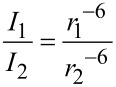


where *I*_1_ and *I*_2_ are intensities of two ROESY peaks and *r*_1_ and *r*_2_ are distances between interacting protons in a given ROESY spectrum.

To see whether this relation can be useful for the study of CD complexes, we performed a molecular dynamics simulation of the aspartame-β-CD complexation from the wider side. From the interproton distances, obtained from the lowest energy frame, we calculated the intensity ratios of all the intermolecular peaks. These peak intensity ratios matched quite well with those calculated from the reported ROESY spectrum of the mixture of aspartame and β-CD [27] but slight refinement of the lowest energy frame conformation gave peak intensity ratios which were in very good agreement. It was observed that peak intensity ratios for each interaction individually, for example, *I*_H-ortho-H-3’_/*I*_H-ortho-H-5’_, or summed intensity ratios (Σ*Ι*_H-3’_/Σ*Ι*_H-5’_) matched very well with experimental ROESY intensities.

**Figure 10:**
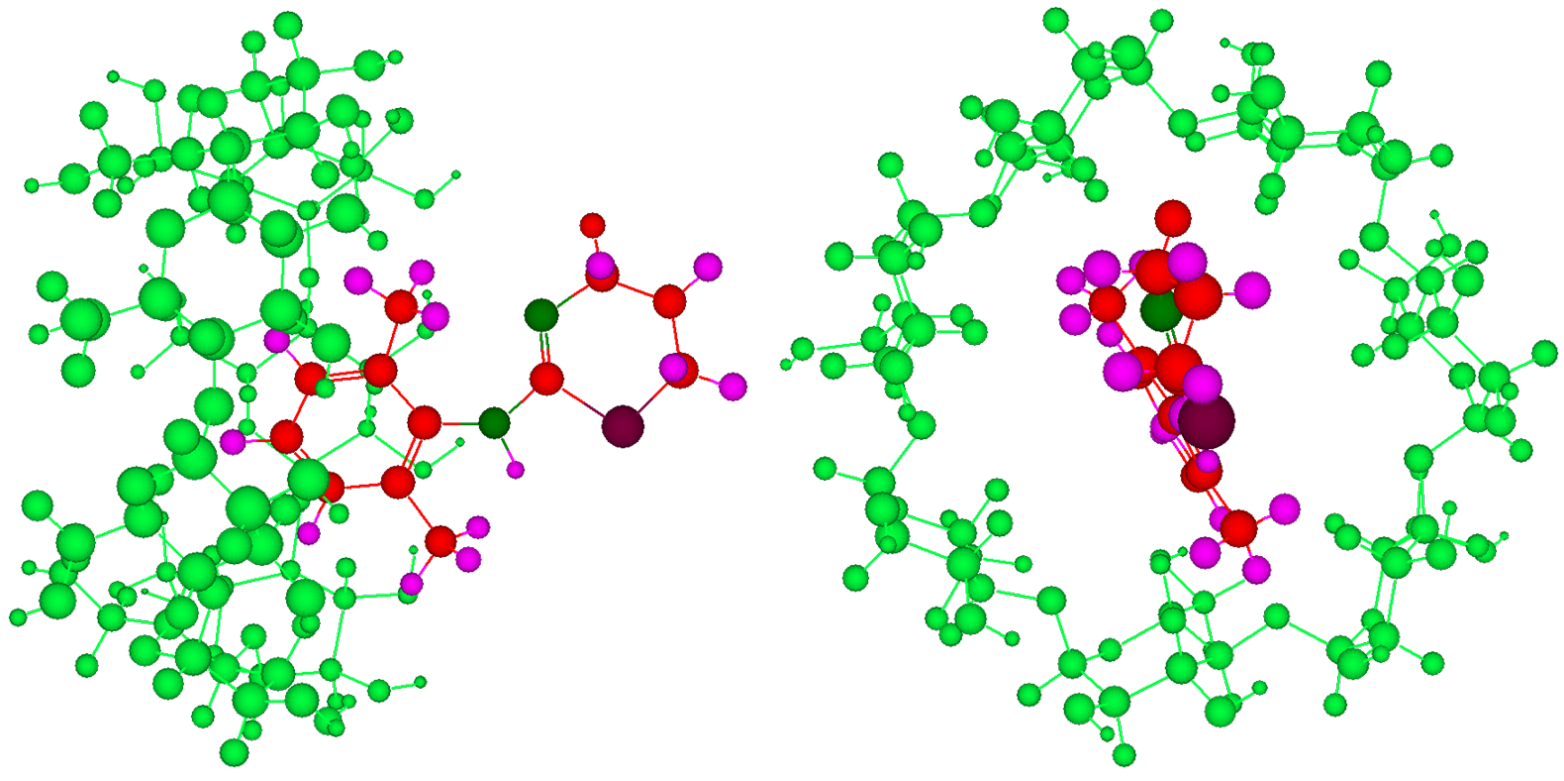
Side and front views of the proposed conformation of the β-CD-xylazine complex.

We then calculated the summed peak intensity ratio Σ*Ι*_H-1_/Σ*Ι*_H-2_ for two frames from the narrow side (Frame 105 and 118) and one frame from the wider side (Frame 132) obtained in molecular dynamics simulations. The interproton distances between aromatic protons of xylazine and cavity protons were obtained for the frames to be studied. All the H–H distances of each aromatic proton with seven H-3’ and seven H-5’ protons were obtained and their referenced peak intensity ratios (*I*_H-1-H-3’_, *I*_H-1-H-5’, _*I*_H-2-H-3’, _*I*_H-2-H-5’_) were calculated using Equation 2 which were then summed to give referenced Σ*I*_H-1-H-3’_, Σ*I*_H-1-H-5’,_ Σ*I*_H-2-H-3’,_ Σ*I*_H-2-H-5’_. The summation of Σ*I*_H-1-H-3’_ and Σ*I*_H-1-H-5’,_ as well as Σ*I*_H-2-H-3’,_ Σ*I*_H-2-H-5’_ gave the total referenced *I*_H-1_ and *I*_H-2_, respectively, from which the ratio was calculated and the results are given in Table 1. The peak intensity ratio of H-1 and H-2 with cavity protons is closest for lowest energy frame from wider side suggesting that this must be the averaged ensemble conformation of the β-CD-xylazine complex (Figure 10). It must be mentioned that the potential energy for narrow side entry is lower than for the wider side and so it must be favored. But, unlike molecular dynamics simulations explicitly in water where contacts outside the cavity are self-compensative, the outside contacts in vacuum also contribute to the total energy and thus energy does not necessarily reflect the complexation energy.

**Table 1:** Peak intensity ratios calculated from ROESY spectrum (experimental) and from conformations obtained by molecular dynamics simulations.

	Potential energy (kcal/mol)	*I*_H-1_ / *I*_H-2_

Experimental	–	4.7
Frame 105 (NS)	97.83	8.7
Frame 118 (NS)	97.37	10.2
Frame 132 (WS)	100.73	5.0

## Conclusion

The results of the study demonstrate that by a combination of quantitative ROESY analysis and molecular dynamics in vacuum, highly accurate structural information regarding the whole conformation of the inclusion complex can be obtained which otherwise only confirms the inclusion of the guest in the cavity. Studies on the determination of the absolute configuration of cyclodextrin complexes by quantitative use of ROESY data are in progress.

## Supporting Information

File 1 The zip-archive contains an xls-file with the molecular dynamics wide side-energy-time plot, a molecular dynamics wide side trajectory in c3d format and the proposed beta-cyclodextrin-xylazine complex in c3d format.

## References

[1] Steed J W, Atwood J L (2009). Supramolecular Chemistry.

[2] Szejtli J (1998). Chem Rev.

[3] Dodziuk H (2006). Cyclodextrins and Their Complexes: Chemistry, Analytical Methods, Applications.

[4] Lipkowitz K B (1998). Chem Rev.

[5] Ivanov P M, Salvatierra D, Jaime C (1996). J Org Chem.

[6] Amato M E, Lipkowitz K B, Lombardo G M, Pappalardo G C (1998). Magn Reson Chem.

[7] Bispo de Jesus M, Pinto L M A, Fraceto L F, Takahata Y, Lino A C S, Jaime C, de Paula E (2006). J Pharm Biomed Anal.

[8] Raffaini G, Ganazzoli F, Malpezzi L, Fuganti C, Fronza G, Panzeri W, Mele A (2009). J Phys Chem B.

[9] Raffaini G, Ganazzoli F (2007). J Inclusion Phenom Macrocyclic Chem.

[10] Demarco P V, Thakkar A L (1970). J Chem Soc D.

[11] Rekharsky M V, Goldberg R N, Schwarz F P, Tewari Y B, Ross P D, Yamashoji Y, Inoue Y (1995). J Am Chem Soc.

[12] Moozyckine A U, Bookham J L, Deary M E, Davies D M (2001). J Chem Soc, Perkin Trans 2.

[13] Nakajima T, Sunagawa M, Hirohashi T, Fujioka K (1984). Chem Pharm Bull.

[14] Pose-Vilarnovo B, Perdomo-López I, Echezarreta-López M, Schroth-Pardo P, Estrada E, Torres-Labandeira J J (2001). Eur J Pharm Sci.

[15] Salvatierra D, Jaime C, Virgili A, Sánchez-Ferrando F (1996). J Org Chem.

[16] Loukas Y L (1997). J Pharm Pharmacol.

[17] Benesi H A, Hildebrand J H (1949). J Am Chem Soc.

[18] Scott R L (1956). Recl Trav Chim Pays-Bas.

[19] Neuhaus D, Williamson M P (1989). The nuclear Overhauser effect in structural and conformational analysis.

[20] Ali S M, Khan S, Crowyn G (2012). Magn Reson Chem.

[21] Available from: http://www.ccdc.cam.ac.uk

[22] Butts C P, Jones C R, Towers E C, Flynn J L, Appleby L, Barron N J (2011). Org Biomol Chem.

[23] Hu H, Krishnamurthy K (2006). J Magn Reson.

[24] Jones C R, Butts C P, Harvey J N (2011). Beilstein J Org Chem.

[25] Macura S, Farmer B T, Brown L R (1986). J Magn Reson.

[26] Bodenhausen G, Ernst R R (1982). J Am Chem Soc.

[27] Sohajda T, Bénia S, Varga E, Iványi R, Rácza A, Szente L, Noszál B (2009). J Pharm Biomed Anal.

